# Is implicit Level-2 visual perspective-taking embodied? Spontaneous perceptual simulation of others’ perspectives is not impaired by motor restriction

**DOI:** 10.1177/17470218221077102

**Published:** 2022-02-25

**Authors:** Eleanor Ward, Giorgio Ganis, Katrina L McDonough, Patric Bach

**Affiliations:** 1 School of Psychology, University of Plymouth, Devon, UK; 2 University of Aberdeen, Aberdeen, UK

**Keywords:** Perspective-taking, visual perspective taking, mentalising, submentalising, perceptual simulation, navigation, mental rotation, mental imagery, active inference

## Abstract

Visual perspective taking may rely on the ability to mentally rotate one’s own body into that of another. Here, we test whether participants’ ability to make active body movements plays a causal role in visual perspective taking. We utilised our recent task that measures whether participants spontaneously represent another’s visual perspective in a (quasi-)perceptual format that can drive own perceptual decision making. Participants reported whether alphanumeric characters, presented in different orientations, are shown in their normal or mirror-inverted form (e.g., “R” vs. “Я”). Between trials, we manipulated whether another person was sitting either left or right of the character and whether participants’ movement was restricted with a chinrest or whether they could move freely. As in our previous research, participants spontaneously took the visual perspective of the other person, recognising rotated letters more rapidly when they appeared upright to the other person in the scene, compared with when they faced away from that person, and these effects increased with age but were (weakly) negatively related to schizotypy and not to autistic traits or social skills. Restricting participants’ ability to make active body movements did not influence these effects. The results, therefore, rule out that active physical movement plays a causal role in computing another’s visual perspective, either to create alignment between own and other’s perspective or to trigger perspective taking processes. The postural adjustments people sometimes make when making judgements from another’s perspective may instead be a bodily consequence of mentally transforming one’s *actual* to an *imagined* position in space.

Humans effortlessly take others’ perspectives and derive what they can or cannot see, or how a scene looks to them (Flavell et al., 1981). This everyday skill allows people to give a passer-by directions so they can plan a route from their own perspective, or work out whether an oncoming driver has noticed them before safely crossing a road, for example. These abilities to understand how others view the world have been argued to underlie the ability to coordinate actions with others (Freundlieb et al., 2016), and may form the basis of more sophisticated social abilities such as reasoning about others’ beliefs, desires, and goals (Batson et al., 1997; Erle & Topolinski, 2015; Mattan et al., 2016; Tomasello et al., 2005).

Recent work has conceptualised the ability to derive another’s viewpoint onto a scene as a form of perceptual simulation, which inserts the content of another’s perspective into one’s own perceptual processes, as if it were one’s own perceptual input (Kampis et al., 2015; Surtees et al., 2016; Ward et al., 2019; but see Cole & Millett, 2019, for a critical view). Such a (quasi-)perceptual representation could then drive one’s own action and decision-making processes just like own input, explaining the developmental link between visual perspective-taking and higher-level mentalising (Batson et al., 1997; Erle & Topolinski, 2015; Hamilton et al., 2009; Mattan et al., 2016; Tomasello et al., 2005) and its link to joint action (e.g., Freundlieb et al., 2016).

A recent series of studies from our laboratory provided direct evidence that people represent others’ perspectives on an object in a similar way to their own visual perspective (Ward et al., 2019, 2020), and that these (imagined) other-perspectives can drive perceptual decision-making processes in the same way as one’s own perceptual input, similar to other perceptual simulation processes (e.g., see Roelfsema & de Lange, 2016, for a review). Prior studies had already provided evidence for an overlap between one’s own and others’ representations of the world, so that stimulus judgements become harder if another person would make the same judgements differently from their perspective (e.g., Samson et al., 2010; Surtees et al., 2016; Tversky & Hard, 2009; Zwickel & Müller, 2010; Zwickel et al., 2010). Yet, these studies left open whether this interference happens on a perceptual level or a conceptual/response level, or whether it simply indexes the uncertainty when a person becomes aware that others would judge the same stimulus differently than oneself. In addition, questions exist on whether these effects truly reflect perspective-taking, or whether they are perhaps better accounted for by domain-general “submentalising” processes, such as the cuing of attention or a coding in object-centred spatial reference frames (i.e., Conway et al., 2017; Heyes, 2014; Santiesteban et al., 2014).

To reveal whether people have (quasi-)perceptual access to the content of another’s perspective, we tested whether another’s viewpoint facilitates perceptual judgements that would be difficult from their own. We adapted the classic mental rotation task, in which participants simply report, as quickly as possible, whether alphanumeric characters at various orientations are presented in their canonical or mirror-inverted form (e.g., “R” vs. “Я”). The well-known finding is that the time it takes to make these judgements increases linearly the more the characters are rotated away from upright (Shepard & Metzler, 1971), because people first must mentally rotate them back into their canonical orientation before being able to judge them. Here, we used this task to test whether people would spontaneously make these judgements *from the perspective of the other person*, so that they can rapidly judge items that are oriented away from themselves, if they appear upright to the other person. Indeed, participants recognised the items more quickly when an incidentally inserted other person would have a more upright view of the to-be-judged character than them, while judgements that would be more difficult from this other perspective became slower. Moreover, regression analyses showed that recognition times (RTs) across letter orientations increased linearly with the angular disparity of the item not only to the participant’s viewpoint, but also to the other person’s viewpoint, suggesting that participants mentally rotated the items from *their own and the other’s* perspective.

These data provided direct evidence that people can mentally represent the content of another’s viewing perspective in a form that can “stand in” for own visual input and drive subsequent perceptual judgements and mental rotation processes. Importantly, these shifts to the others’ perspective occurred spontaneously, even when the persons in the scene were completely task irrelevant. Further studies showed that the same effects were not present when the person was substituted for an inanimate object (i.e., a lamp that “looks” at the letter as the persons did, Ward et al., 2019), but increased substantially when participants were explicitly asked to take the other person’s perspective. More recent work (Ward et al., 2020) showed that these shifts into the other’s perspective are not sensitive to where this person currently looks but reflect their location in space and which perspectives this vantage point would, in principle, afford (irrespective of where the person actually looks).

An interesting anecdotal observation was that, within these tasks, participants would sometimes inadvertently shift their *actual* position towards the other person’s, angling their head slightly rightwards if another person appeared to the left of the items on the screen, and leftwards if the person appeared to the right. This observation fits with the view that perspective-taking is an “embodied” process (e.g., Kessler & Rutherford, 2010; Kessler & Thomson, 2010; Kessler & Wang, 2012), in which people mentally rotate themselves into the position of the other person. Studies have shown, for example, that explicit perspective taking (i.e., consciously judging how a scene would appear to another person with a different view) takes longer the more another person is rotated from one’s own perspective (Kessler & Rutherford, 2010; Kozhevnikov et al., 2006; Surtees et al., 2013). Similarly, when people physically align their posture with that of another person, judgements from this *other*-perspective become easier, while adopting a misaligned posture makes it harder to take this other person’s perspective. In this view, the subtle adjustment of posture we observed might therefore reflect an epiphenomenal “leakage” from the mental transformation of people’s actual to the imagined other-position, similar to other bodily consequences of motor imagery (Bach, Allami, et al., 2014; Bach et al., 2010; Colton et al., 2018; Jacobson, 1930; Vargas et al., 2004).

Here, we ask whether these bodily movements are not simply bodily signs of a mental perspective transformation, but whether they play a *causal* role in driving the shift to the other person’s perspective. There are two ways in which overt body movements could facilitate judgements from the other person’s perspective. First, several recent proposals from the field of embodied cognition argue that people actively use their own body and the environment to scaffold cognitive judgements (e.g., Glenberg, 2010; Proffitt, 2006 for perspective taking, see Tversky & Hard, 2009). In our case, people could have used the bodily movement to trigger “embodied” processes that allow them to picture the world from another’s perspective. When people grow up, they develop highly automatic processes that allow them to predict the perceptual consequences of their actions (i.e., “forward models,” Blakemore et al., 1999; Miall & Wolpert, 1996), such that they can predict, before the action is completed, which visual (e.g., Hughes & Waszak, 2011), auditory (Kunde et al., 2004), or tactile sensations it will produce (e.g., Morrison et al., 2013; Bach, Fenton-Adams, & Tipper, 2014). In mental rotation tasks, it has been shown, for example, that manual rotations consistent with the speed and direction of mental rotations facilitate faster judgements. These movements appear to directly support the mental rotation, as restricting these movements or asking participants to make different movements interferes with the imagery of finger movements (e.g., Vargas et al., 2004) or mental rotation processes (Wohlschläger & Wohlschläger, 1998). Similar links have been observed for emotion judgements and restrictions of one’s own facial musculature, restriction of hand movements and abstract mathematical relationships (Cook et al., 2012; Neal & Chartrand, 2011; Parsons, 1994), and aesthetic judgements (Woltin & Guinote, 2015). In our task, therefore, people could make subtle overt movements towards the other person’s location for the same purpose: to trigger the very processes that predict the perceptual consequences of how the world would look if these movement had been completed.

A second possibility is that the body movements reflect actual attempts to effectively sample the scenes from the other person’s perspective. Recent proposals from the domain of predictive processing argue that perception is not a passive process, but a process of “active inference” in which people constantly move their bodies (Friston et al., 2009) and their eyes (e.g., Parr & Friston, 2017) to most effectively sample the information that they require for the task, or to fulfil their prior expectations and avoid “surprising” states (Friston, 2010). In our task, the presence of a person on the left or the right might have triggered body movements so that people’s own perspective—and the perceptual input they receive—aligned more closely with that of the other person. For our task, this raises the possibility, therefore, that the measured shifts into the other’s perspective do not reflect changes to participants’ *mental* representation of perceptual input, but a change in the *perceptual* input they receive brought along by the body movements they make, so that they can actually see the item better in orientations that align with the other person’s location.

One effective way to test whether the subtle body movements of participants play a role in perspective taking is by comparing performance in conditions in which these movements are possible and conditions in which they are restricted. As noted above, movement restriction manipulations have long been used to test whether motor processes play a role in cognitive tasks, across a variety of tasks from emotion perception (Neal & Chartrand, 2011), to mathematical reasoning (Cook et al., 2012), to aesthetic judgements (Woltin & Guinote, 2015). In particular, during social perception, restricting people’s mouth movements impairs recognising emotions in others’ faces (e.g., Jospe et al., 2017, 2018; Neal & Chartrand, 2011; Orlowska et al., 2021), presumably because people can no longer match their physical body state to that of the observed person. More generally, restricting hand movements disrupts people’s access to visuospatial content (Cook et al., 2012; Rauscher et al., 1996) and biases them towards visual rather than motor strategies in mental rotation (e.g., Chu & Kita, 2008; Moreau, 2013; Sirigu & Duhamel, 2001). By the same token, restricting one’s head/body movements should disrupt both one’s ability to physically match one’s visual perspective to that of the avatar in the scenes, and one’s ability to trigger “embodied” perspective taking processes that mentally rotate one into the avatar’s body. To the extent that previous perspective taking effects depend on either mechanism, they should be reduced when these movements are restricted.

We gave participants the same mental rotation task as in our previous studies (Ward et al., 2019, 2020) and asked them to report whether alphanumeric characters appearing on a table in front of them in different orientations were presented normally or were mirror-inverted (e.g., “R” vs. “Я”). In some of the trials, a person appeared in the scenes and looked at the items from either the left or the right of the table. This allows us to measure how much faster items are identified when they face the other person (and therefore appear upright to them), compared with facing away from them. The crucial manipulation was that in half of the trials, participants’ movement was restricted using a chinrest. In these trials, they could therefore not adjust their own body movement to either actively sample the scenes from the others’ perspective or to trigger “embodied” perspective taking processes. If movements are causal in creating the shifts to the others’ perspective, then restricting participants’ movement should disrupt perspective taking, and the response time benefits for items easy to recognise for the other person would be reduced or eliminated. If, however, the movements are simply epiphenomenal “leakage” of mental rotations into the other person’s body, then preventing these movements should have no effect.

A second goal of the current study was to explore whether, and, if so, which, individual differences determine the tendency to spontaneously take another’s visual perspective. Testing for such potential relationships is important because they provide insights about the role the measured processes play in everyday life, and how or whether they are related to individuals’ higher-level social interaction skills. Several candidate characteristics exist. First, prior work suggests that individuals with schizophrenia are impaired in social interactions and understanding (for a review, see Brüne, 2005) and they have specific difficulty in tasks requiring mentalising (e.g., Langdon & Coltheart, 1999, 2001; Langdon et al., 2001) and/or own body spatial transformations (Mohr et al., 2006). We therefore tested whether participants’ tendency to spontaneously compute the other’s visual perspective (as measured in our task) is negatively related to schizotypal traits, assessed by the Schizotypy Questionnaire (STQ; Claridge & Broks, 1984). Similarly, autism spectrum conditions have long been associated with problems in Theory of Mind in general (Frith et al., 1991; for a review, see Hamilton, 2009) and perspective taking in particular (Hamilton et al., 2009). We therefore also gave the autism quotient (AQ; Baron-Cohen et al., 2001) to all participants, to ascertain whether autistic-like traits in the neurotypical population predict spontaneous perspective-taking. Note that autism has been specifically linked to difficulties in selecting, not computing, another’s visual perspective (e.g., Qureshi et al., 2010; Ramsey et al., 2013; Schwarzkopf et al., 2014). If true, no relationships are expected, as our task was designed to measure spontaneous perspective computation, not intentional perspective selection. Finally, we tested for the proposed link between spontaneous perspective taking and general mentalising/social interaction skills (e.g., Batson et al., 1997; Erle & Topolinski, 2015; Mattan et al., 2016; Tomasello et al., 2005), using the Interpersonal Reactivity Index (IRI; Davis, 1983). A link between perspective taking and the IRI and various measures of perspective taking has been reported before (e.g., Level 1 VPT, Bukowski & Samson, 2017; emotional perspective taking, Trilla et al., 2021).

## Method

### Participants

A total of 79 naive participants (59 women, 1 non-binary gender) were recruited via the University of Plymouth student participation pool. All participants were adults (age range 18–35) and gave written informed consent according to the declaration of Helsinki. Approval was obtained from the University of Plymouth Ethics Committee. Seven additional participants were not analysed due to malfunctioning response recording. Participants received course credit as compensation. After exclusion (error > 20%), the remaining 61 participants (46 women, 1 non-binary gender; mean age: 20.5 years, range: 18–35) provide 80% power to detect effects in the range of *d* = .32. Prior work on this paradigm (Ward et al., 2019) has revealed that effect sizes are substantially larger (.747 < *d* < 1.08 for the main perspective taking effect). For correlations with measures of individual differences, the 61 participants provide 80% power to detect correlation coefficients of *r* = .25 (two-tailed), or *r* = .23 (one-tailed).

### Apparatus, stimuli, and procedure

All experiments were conducted in the behavioural testing lab space of the University of Plymouth. The experiments were administered using Presentation® software (Version 18.0, Neurobehavioral Systems, Inc., Berkeley, CA, www.neurobs.com). Stimuli were presented on a 19-in. LED computer monitor (resolution: 1,900 x 1,200; refresh rate: 60 Hz). Responses were made on a standard computer keyboard with UP, DOWN, and SPACE keys as active response keys. Red and green stickers were positioned on the DOWN and UP keys, respectively. A standard chinrest was provided for participants, fixed with a screw clamp central to the computer monitor at a distance of 60 cm, and a height of 30 cm from the desk surface. Participants’ actual body or head movements were not recorded.

**Figure 1.**
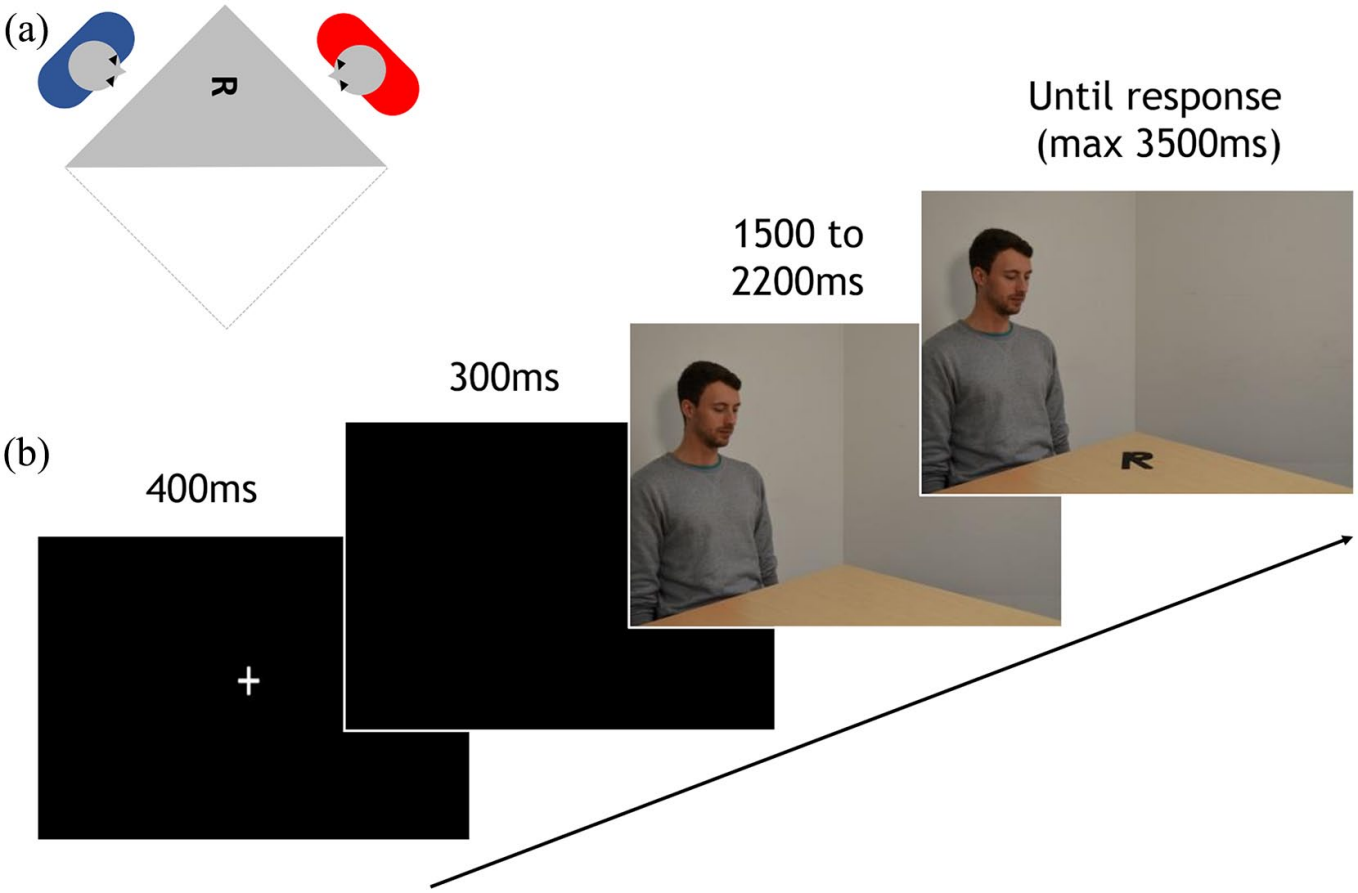
(a) Scene set-up and (b) schematic of the trial sequence. Panel A shows the position of the person in the scene relative to the participant and the character on the table, producing a viewpoint rotated by approximately 90° relative to the participant. Panel B shows the timing of the trial sequence. First, participants viewed a fixation cross followed by a blank screen. The next frame showed a male (pictured above) or female actor positioned either to the left or the right of the table. After a random period of 1,500–2,200 ms, an alphanumeric character appeared on the table either in its canonical or mirror-inverted form. Participants responded with a button press to indicate whether they thought the letter was normal or mirrored. In half of all trials, participants’ movement was restricted using a standard chinrest.

Participants sat upright facing the screen at a distance of approximately 60 cm and were given written and verbal instructions. They were given examples of the rotated items that would appear on the screen and completed eight training trials that were identical to the main experiment (Figure 1). Each trial (total trials = 572) started with a fixation cross displayed for 400 ms, followed by a blank screen for 300 ms. The subsequent stimulus sequence included two frames, measuring 33.4° × 23.5° of visual angle, presented without interstimulus interval. The first frame was presented for a random period between 1,500–2,200 ms. In one-third of the trials, it showed a view onto a corner of a square table in a grey room. The remaining trials showed a person sitting behind the same square table, gazing at the centre of the table. The person could either be male or female and sat either on the left or right side of the table in an equal number of trials.

The second frame in the sequence was identical to the first frame, but now 1 of 48 possible items appeared on the table, at the location on the table the on-screen person was gazing at. This item was one of three alphanumeric characters (4, P, or R), presented either in the canonical version or mirror-inverted about their vertical axis, in one of eight orientations (0°, 45°, 90°, 135°, 180°, 225°, 270°, 315°, with 0° denoting the upright canonical orientation and angles increasing in a counter-clockwise fashion) relative to the participant. The characters always appeared in the same position on the table, halfway between the outward corner of the table and its centre, such that the persons to the left and right would gaze at the table from roughly 90° and 270°, respectively (perpendicular to the viewpoint of the participant), as at these angles the character’s angular disparities from the participant and the other person were statistically orthogonal across conditions. Rotation of the alphanumeric characters occurred around their centre point. This frame remained on the screen until a response was made to a maximum duration of 3,500 ms. Participants were asked to judge whether each character was presented in its canonical or mirror-inverted form. Participants responded using their right hand by pressing the green key to indicate a canonical item and the red key to indicate a mirrored item. Response times were measured relative to item onset.

The trials were divided into four blocks of 144 trials each. Half were completed using a chinrest (height 30 cm from desk, 60 cm from screen) to restrict motion, and the remaining half of the trials were completed without a chinrest, in an ABAB order, counterbalanced across participants. The presented stimuli were pseudorandomised across blocks, such that all possible combinations of actor-location/item/presentation/orientation were shown in both the no-movement (chinrest) and no-free-movement (no-chinrest) condition throughout the experiment. In both conditions the viewing distance from head to screen was approximately 60 cm, as in all previous experiments.

### Quantification and statistical analysis

Data (pre-)processing and analysis were identical to Ward et al. (2019, 2020) and conducted in Microsoft Excel (2010) and JASP (2018). Violin plots were created using Raincloud Plots (Version 1; Allen, Podiaggi, Whitaker, et al., 2019). Power analyses were conducted in G*Power (Version 3.1; Faul, Erdfelder, Lang & Buchner, 2007).

Dependent measures were the RTs (measured from item onset) for each character orientation (0°, 45°, 90°, 135°, 180°, 225°, 270°, 315), depending on person location (no-person, person-left, person-right) and movement condition (free-movement, no-movement). Analogous analyses of error rates were also conducted to rule out speed/accuracy trade-offs. In both conditions, error rates numerically followed the pattern of the main RTs but did not show statistically reliable differences (Table 1).

**Table 1.** Means (*M*) and standard deviations (*SD*) for the left/right and towards/away biases in error rates in all conditions. Forward/away and left/right biases were calculated analogously as for the recognition times.

	Towards/away bias			Left/right bias		
Condition	Person-left *M* (*SD*)	Person-right *M* (*SD*)	No-person *M* (*SD*)	Person-left *M* (*SD*)	Person-right *M* (*SD*)	No-person *M* (*SD*)
All	−.023 (.02)**	−.02 (.02)**	−.021 (.02)**	−.005 (.015)*	.002 (.016)	−.001 (.014)
Free-movement	−.022 (.02)**	−.017 (.02)**	−.019 (.03)**	−.006 (.019)*	.000 (.022)	.002 (.021)
No-movement	−.024 (.02)**	−.022 (.02)**	−.024 (.02)**	−.005 (.021)*	.003 (.021)	−.004 (.017)

* *p* < .05. **p < 001.

To quantify changes in RTs when the characters either faced the participant (i.e., was seen in its canonical orientation from the perspective of the participant) or the other person in the scenes, we derived two analogous and statistically independent summary measures, as in our previous work (Ward et al., 2019). The first summary measure *towards/away-bias* indexes to what extent characters were recognised faster the more they faced towards the participant (0°) rather than away from them (180°), separately for each participant and each condition (no-person free-movement, person-left free-movement, person-left no-movement, person-right free-movement, person-right no-movement, and no-person no-movement). This measure therefore quantifies the mental rotation effect (Shepard & Metzler, 1971). The second summary measure (left/right bias) indexes how much faster characters are recognised the more they are oriented towards the left (270°) rather than right (90°), or vice versa. This allows us to test whether a participant spontaneously takes the actor’s perspective, as the left/right bias—how much faster left-oriented than right-oriented letters are judged—should then depend on whether the actor sits on the left or the right.

The contribution of each character orientation to the two summary measures was derived by treating each participant’s RT for this character orientation as a vector in a coordinate system, with the RT providing the distance from the origin and the rotation angle the polar angle. A character orientation’s contribution to the towards/away bias was then derived simply from the RTs multiplied with the negative of the cosine of the orientation angle. As a result, characters contribute negatively the more they face the participant (315°, 0°, 45°) and positively they more they are oriented away from them (225°, 180°, 135°). Similarly, the contribution of a character’s orientation to the left/right bias was calculated as the RT multiplied with the sine of the orientation angle. Character orientations contribute positively the more they face to the left (45°, 90°, 135°) and negatively the more they face to the right (225°, 270°, 315°). This procedure effectively maps the changes evident in the radar plots for each angle onto two orthogonal and statistically independent summary measures, so that they can be compared across conditions without accruing alpha inflation due to multiple testing, which would result if each of the eight angles were compared separately.

By averaging these values, separately for each summary measure, participant and condition (no-person, person-left, person-right), we are able to calculate, first, whether characters were recognised faster the more they appear in the canonical orientation to the participant (negative values on the towards/away bias) compared with when they are oriented away (positive values), reflecting the expected mental rotation effect. Similarly, they allowed us to calculate to what extent items were recognised faster the more they were oriented leftwards and therefore would appear in their canonical orientation to a person sitting to the left (positive values on the left/right bias) rather than rightwards, where they would appear in their canonical orientation to a person sitting on the right (negative values). We were then able to determine whether this left/right bias changed depending on whether another person was presented in the scenes and on whether the person was on the left or on the right.

The crucial comparison is the difference between the left/right biases in the person-left and person-right conditions, which describes how much faster letters are recognised when rotated left than right, depending on whether the other person is sitting to the left or right. Note that the direct comparison of the person-left and person-right conditions is statistically identical to the comparison of how much person presence shifts mental rotation performance in the person-left and person-right conditions relative to the no-person baseline (i.e., how much person presence shifts RTs away from 0° towards either 90° or 270°), as this would involve subtracting the same baseline value from each of the two conditions for each participant, and would therefore not affect the absolute difference between them.

### Across-participant regression analyses

In prior work, the mental rotation effect is sometimes characterised in terms of separate linear regressions of an item’s RT to its angular disparity relative to the participant, for each participant separately (Shepard & Metzler, 1971). The results reveal linear increases with increasing angular disparity for the large majority of participants. Here, we used this analysis model to test whether an item’s RTs can be described, on a single participant basis, as a linear increase of the character’s angular disparity *both* to the participant and to the other person. To this end, we entered each participant’s item mean RTs for each character orientation in the person-left and person-right condition as dependent variable in a single multiple regression, for each participant separately, with the item’s angular disparity to the participant and to the other person as two statistically independent predictors. This analysis provides statistically independent regression coefficients for both predictors—angular disparity to participant and the other person—for each participant separately. We report mean across-participant regression coefficients for each of these two predictors and compare them with *t*-tests against zero.

### Individual differences measures

All participants were given three paper questionnaires after the computer task. First, the AQ (Baron-Cohen et al., 2001) consists of 50 questions assessing social skills (e.g., I enjoy social occasions), attention to detail (e.g., I often notice small sounds when others do not), attention switching (e.g., I prefer to do things the same way over and over again), communication (e.g., I enjoy social chit-chat), and imagination (e.g., I find making up stories easy). The overall score gives a measure of autistic traits, where numerically high scores indicate high levels, and scores at the lower end indicate lower levels of autistic traits. Responses are recorded using a 4-point Likert-type scale, with the options *definitely agree*, *slightly agree*, *slightly disagree*, and *definitely disagree*. Prior validation studies (e.g., Baron-Cohen et al., 2001; Wakabayashi et al., 2006) show moderate to high internal consistency (Cronbach’s alpha = .63–.77) and high test–retest reliability (*r* = .70), in both autistic and neurotypical samples.

Second, the IRI (Davis, 1983) is a 28-item questionnaire measuring empathy, comprising the four subscales measuring perspective taking (e.g., I sometimes try to understand my friends better by imagining how things look from their perspective), empathic concern (e.g., I am often quite touched by things that I see happen), fantasy (e.g., I daydream and fantasise, with some regularity, about things that might happen to me), and personal distress (e.g., When I see someone who badly needs help in an emergency, I go to pieces). Responses are made on a 5-point Likert-type scale ranging from *does not describe me well* to *describes me very well*. Numerically high scores indicate high levels of empathy, while lower scores indicate low levels of empathy. Prior validation studies show moderate to high internal consistency (Cronbach’s alpha = .73–.83; De Corte et al., 2007) and high test–retest stability (intraclass correlation coefficients = .71–.86; Gilet et al., 2013).

Finally, the STQ (Claridge & Broks, 1984) is a short measure of schizotypal personality traits, and consists of two scales, corresponding to the distinction made in the *Diagnostic and Statistical Manual of Mental Disorders, Third Edition* (*DSM*-III; American Psychiatric Association, 1980) between schizotypal personality disorder (STA scale) and borderline personality disorder (STB scale). Simple “yes/no” responses are made to questions targeting schizophrenic-like features (e.g., Do you ever suddenly feel distracted by distant sounds that you are not normally aware of?), and borderline-personality traits (e.g., Do you at times have an urge to do something harmful or shocking?), scoring 1 for *yes* responses, and 0 for *no* responses. Numerically high scores indicate higher levels of schizotypy, while lower scores indicate lower levels. Here, we were interested specifically in schizotypal traits, therefore only responses for questions 1–37 in the STA part of the STQ are collected and reported in this study.

We tested whether either of these individual difference measures predicts people’s spontaneous tendency to take the other person’s perspective. This tendency is indexed by the difference between left/right biases when a person is sitting on the left compared with when they are sitting on the right, reflecting how much faster/slower item recognition the more items are oriented towards/away from the other person. We therefore calculated this difference for each participant separately by subtracting the mean left/right-bias value for a person sitting on the right from the value for a person sitting on the left. Scores for the AQ (Baron-Cohen et al., 2001), the IRI (Davis, 1983), and the STQ (Claridge & Broks, 1984) were then entered as predictor variables, and perspective taking scores as the dependent variable, into a multiple linear regression model.

## Results

As in our prior work (Ward et al., 2019, 2020), erroneous responses (8% on average) were excluded from the analysis of RTs, as well as trials with RTs longer than 2,000 ms, or shorter than 150 ms.

### Mental rotation

We first confirmed that our data replicate the known mental rotation effect (Shepard & Metzler, 1971), where RTs increase linearly with the item’s angular disparity to the participant. We first derived the overall (across conditions) towards/away bias, indexing in milliseconds how much more slowly items are identified the more they are rotated away from the participant compared with towards them, and compared it with a simple *t*-test against zero. This towards/away bias was positive in all conditions, *M* = 54.14; *SD* = 22.09, *t*(60) = 19.14, *p* < .001, *d* = 2.45, *BF_10_* = 2.124e + 24, showing, unsurprisingly, that items are identified more quickly the more they are oriented towards the participant. We further confirmed this mental rotation effect by regressing each item’s RT to the expected linear increase with angular disparity, as in prior research (Shepard & Metzler, 1971; Ward et al., 2019), revealing positive slopes in all bar one participant, mean β = 1.5; *t*(60) = 23.34, *p* < .001, *d* = 2.99, *BF_10_* = 3.124e + 28.

We then verified that this overall mental rotation effect was not affected by person presence and the chinrest manipulation. As the actors would sit at 90° and 270° angle to the participant, and their location was therefore orthogonal to the towards/away axis, we did not expect that person presence/location would affect the overall mental rotation effect. Indeed, a 2 x 3 analysis of variance (ANOVA) on the towards/away biases across conditions with the factors movement (no-movement, free-movement) and location (person-left, person-right, no-person) did not reveal any significant main effects or interactions, *F* < 1, *BF*_10_ < .155 for all. When conditions were analysed separately, decisive evidence of slower recognition for turned away items was present in all conditions, *t*(60) > 12.99, *p* < .001, *d* > 1.7, *BF*_10_ > 1.188e + 16, for all.

### Perspective taking

**Figure 2.**
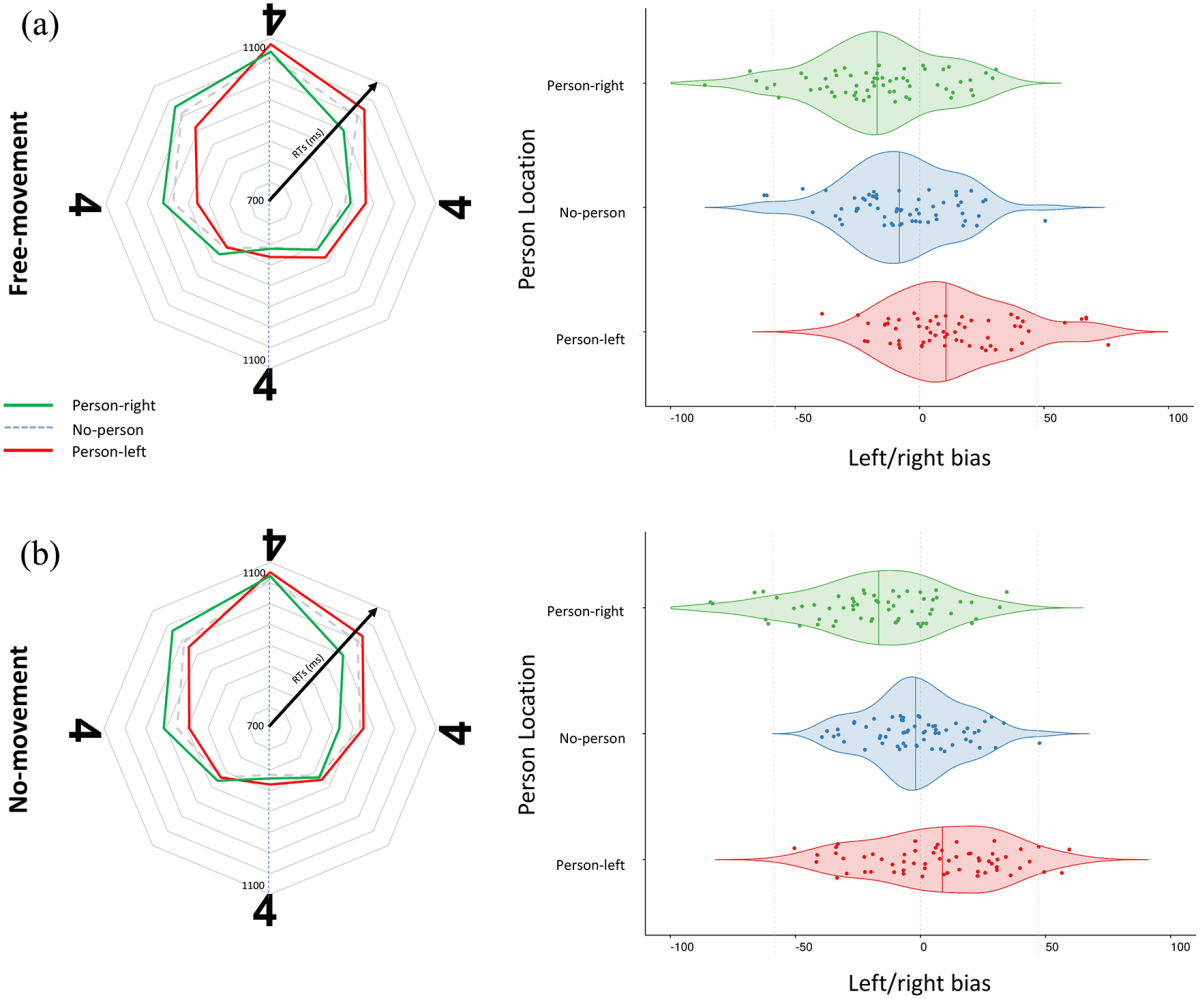
Results for both free-movement and no-movement conditions: movement restriction does not impede visual perspective taking. (a) Free-movement condition. Left panel: mean recognition times (ms) to correctly classify items as canonical or mirror-inverted in each of the eight orientations depending on whether the person was absent (dotted line), sitting on the left (red), or sitting on the right (green). Right: violin charts showing the left/right bias, which marks how much more quickly the participant classified items oriented towards the left than the right, depending on whether the other person in the scene was sitting on the right (top), was absent (middle), or was sitting on the left (bottom). (b) No-movement condition. Left: mean recognition times (ms) for mirror-inverted/canonical judgements as described for (a). Right: left/right bias when participants’ movement was restricted using a chinrest, as described for (a).

The main question was whether people would spontaneously take the perspective of the other person in the scenes, such that items were recognised faster when oriented towards compared with away from this other person, and whether this effect, in turn, was determined by whether participants were able to physically align their posture with the actors in the scenes. We therefore derived, for each participant and condition separately, the left/right bias, which indexes how much faster left-oriented items are identified compared with right-oriented items (Figure 2). Here, positive values indicate faster RTs for left-oriented items (upright to person sitting on the left) and negative values indicate faster recognition of items oriented to the right (upright to a person seated on the right).

These left/right biases were entered into a 2 × 2 ANOVA, with location (person-left, person-right) and movement (no-movement, free-movement) as within subject factors. Replicating our prior work, this analysis revealed decisive evidence of a main effect of location, *F*(1,60) = 50.556, *p* < .001, η_*p*_^2^ = .457, *BF_10_* = 2.240e + 14. As in our prior work (Ward et al., 2019), left/right biases were more negative (indexing faster recognition of rightwards- than leftwards-oriented letters) when someone was sitting on the right, and more positive (indexing faster recognition of leftwards- than rightwards-oriented letters) when someone was sitting on the left, confirming that the presence of another person facilitates faster judgements when items are seen as upright from the position of this other person.

The predicted interaction of location and movement, *F*(1,60) = .689, *p* = .41, η_*p*_^2^ = .011, *BF_10_* = 1.196, was not significant, indicating that people spontaneously simulate the visual perspectives of the inserted persons, even when they are unable to physically align themselves with their position in space. Direct comparisons for left/right bias revealed reliable differences for person-left and person-right locations in both the free-movement condition, *t*(60) = 6.81, *p* < .001, *d* = .87, *BF_10_* = 2.367e + 6, and in the no-movement condition, *t*(60) = 5.07, *p* < .001, *d* = .65, *BF_10_* = 3916.17. Next to this, the analysis only revealed an unpredicted and theoretically irrelevant main effect of movement, so that RTs were generally faster in the free movement condition, but Bayesian analyses revealed this effect to be negligible, *F*(1,60) = 4.82, *p* = .031, η_*p*_^2^ = .074, *BF_10_* = .381.

### Regression analysis

As in our previous studies, we tested whether RTs could be described as independent linear increases depending on an item’s angular disparity to the participant as well as the other person, by using both disparities as orthogonal predictors in a single simple regression model, for each participant and condition separately (and then comparing them against zero). Overall, these revealed very strong evidence for independent contributions of both the angular disparity to the participant, mean β = 1.39, *t*(60) = 19.181, *p* < .001, *d* = 2.46, *BF_10_* = 1.189e + 24, and to the other person, mean β = .38, *t*(60) = 7.209, *p* < .001, *d* = .92, *BF_10_* = 1.000e + 07, showing that RTs can be described by independent mental rotation functions from one’s own and the other person’s perspective.

To test how these linear relationships were affected by participants’ ability to move freely, each participants’ beta estimates were entered into a 2 × 2 repeated measures ANOVA with movement (free-movement, no-movement) and viewpoint (self, other) as within-subject factors. As expected, this analysis provided decisive evidence for a main effect of viewpoint, *F*(1,60) = 129.57, *p* < .001, η_*p*_^2^ = .68, *BF_10_* = 2.117e + 32, showing that the angular disparity towards the participants determined RTs to a stronger extent that angular disparity to the other person. As in the main analysis, it provided considerable evidence against any influence of the ability to move freely on the linear relationships. There was neither a main effect of movement, *F*(1,60) = .615, *p* = .436, η_*p*_^2^ = .010, *BF_10_* = .154, nor an interaction of movement and perspective, *F*(1,60) = .016, *p* = .9, η_*p*_^2^ = .00. *BF_10_* = .141. Thus, there was neither an overall change in how strongly angular disparities to the participant and the other person determined RTs, nor a specific change in the contribution of either the angular disparity to the participant and the other person. Indeed, in the no-movement trials, both the angular disparity away from upright to the participant, mean β = 1.37, *t*(60) = 16.01, *p* < .001, *d* = 2.05, *BF_10_* = 1.632e + 20, and to the actor, mean β = .35, *t*(60) = 5.02, *p* < .001, *d* = .64, *BF_10_* = 3,507.56, determined RTs. The same was also true for free-movement trials, where angular disparity to the participant, mean β = 1.41, *t*(60) = 17.426, *p* < .001, *d* = 2.23, *BF_10_* = 9.920e + 21, and to that of the other person, mean β = .40, *t*(60) = 6.87, *p* < .001, *d* = .88, *BF_10_* = 2.818e + 06 provided reliable contributions to RTs.

### Relationships to individual differences

A second goal of the study was to test how individual differences in the tendency to judge the items from other’s perspective is related to individual differences in schizotypy, autistic traits, and reactivity in social interactions. We therefore correlated each participant’s spontaneous perspective taking score (the difference in the left/right bias when the other person was sitting on the left or the right) separately with each of their three questionnaire scores and their age, across all conditions. Other correlations of potential interest are reported in Table S1.

We first correlated participants’ age with spontaneous perspective taking scores measured in our task, replicating our prior finding (Ward et al., 2019) that people take another’s perspective more as they increase in age, *r* = .38, *p* = .003, *BF_10_* = 13.52. We then tested whether perspective taking was negatively correlated with participants’ self-reported measures of schizotypy as seen in prior work (Langdon & Coltheart, 2001; Langdon et al., 2001). The results indeed revealed a negative relationship between participants’ STQ scores and their spontaneous perspective taking scores, replicating this finding, *r* = −.26, *p* = .044, *BF_10_* = 1.166. Note that here *BF* is below 3 and close to 1, indicating that this may be a spurious effect. We then correlated spontaneous perspective taking scores against IRI scores (*M* = 68.45; *SD* = 11.34) and AQ scores (*M* = 16.64; *SD* = 6.29) giving a measure of the relationship between perspective taking and social ability. Neither revealed a reliable relationship, *r* < .08, *p* > .543, *BF_10_* < .191, for all. No correlations were observed even when correlations were computed separately for each of the questionnaires’ subscales (IRI, all *r* < .102; AQ, all *r* < .175).

Multiple linear regressions analyses were conducted to test whether these questionnaire scores and participants’ age were reliable predictors of perspective taking. Using the enter method, all variables were hierarchically entered into the model individually, revealing that when all variables were included, the model reliably predicted spontaneous perspective taking score, *R*^2^ = .24, *F*(4,53) = 4.059, *p* = .006. With all variables included, beta coefficients confirmed that both age, *β* = .376, *t* = 3.013, *p* = .004, and STQ score, *β* =−.268, *t* =−2.154, *p* = .036, provided reliable contributions to the model, while AQ score, β = .093, *t* = .679, *p* = .5, and IRI score, β = .094, *t* = .716, *p* = .477, did not.

The addition of STQ scores increased the predictive power of a model containing only age as a predictor by 6%, *F*(1,55) = 4.210, *p* = .045, but the individual addition of AQ and IRI scores as predictors did not improve the model, R^2^change < .07%, *F*(1,54/53) < .513, *p* > .477 for all, further confirming that AQ and IRI scores are unrelated to our measure of spontaneous visual perspective taking.

## Discussion

We tested whether people’s tendency to spontaneous take another’s visual perspective depends on their ability to make active body movements to physically align one’s own perspective with that of another. In a version of our recent task (Ward et al., 2019, 2020), we asked participants to judge the presentation (mirror-inverted or canonical) of alphanumeric characters on a table, shown at varying orientations. Between trials, an incidentally presented person appeared either to the left or the right of the item. The results replicated, first, the well-established mental rotation effect (e.g., Shepard & Metzler, 1971), with RTs increasing linearly the more items were rotated away from the participant’s own viewing perspective, in line with the idea that the items first must be mentally rotated back into their canonical (upright) orientation before they can be judged. The results also replicated our finding (Ward et al., 2019, 2020) that participants spontaneously draw on the other person’s perspective to make these judgements. Participants recognised items oriented away from themselves more quickly when the items would appear upright to the other person in the scenes, and more slowly when the items are even further rotated away from the other person. Thus, leftward-oriented items were recognised more quickly when another person saw the letter from the left than the right, and vice versa for rightward-oriented letters. Finally, regression analyses replicated the finding that RTs increased linearly not only with the item’s angular disparity to the participant, but also to the other person, suggesting a mental rotation from their own and the other’s perspective. Together, these data therefore confirm that people spontaneously represent other’s visual perspectives in a manner that can “stand in” for own perceptual input. Once represented in such a manner, the others’ view on the task relevant item can drive item recognition and mental rotation processes like one’s own perceptual input, facilitating judgements that would be easier from this other-perspective and slowing those down that would be more difficult.

The crucial question was whether these spontaneous shifts into the other’s perspective depend on people’s ability to shift their own body posture into the other’s position, either because such movements physically more closely align one’s own viewpoint with that of another, or because they trigger “embodied” rotation processes into the other’s location in space. Prior research has shown that several cognitive processes, from mental rotation (Wohlschläger & Wohlschläger, 1998) to emotion recognition (Neal & Chartrand, 2011) or mathematical reasoning (Cook et al., 2012) are supported by the body movements people make at the same time, with performance decreasing if these movements are restricted or not compatible with the mental operation. Movement restriction in particular has been shown to disrupt people’s ability to read emotion from others’ faces (Jospe et al., 2017, 2018; Neal & Chartrand, 2011), their use of motor strategies in mental rotation tasks (Moreau, 2013; Sirigu & Duhamel, 2001), and their general access to visuospatial content (Chu & Kita, 2008; Cook et al., 2012; Rauscher et al., 1996). We did not, however, find similar disruptive effects in our spontaneous visual perspective taking task. When participants’ movements were restricted with a chinrest, the shifts into the others’ perspective were just as strong as when participants were free to move, showing that people’s ability to derive how a scene looks to another does not rely on the ability to *physically* move one’s head or body.

These findings show, first, that visual perspective taking, as measured in our task (Ward et al., 2019), cannot simply be explained as a consequence of participants’ physical alignment with the persons on the screen, which could potentially make item recognition easier. Several proposals argue that perception is not a passive process, but a process of “active inference” in which people actively move their body to better sample the information required (e.g., Friston, 2010). Our data strongly rule out that, in our task, simulation of others’ perspectives is achieved by bringing one’s own perspective into *physical* alignment with that of the other person so that the actual input from the stimulus changes as a consequence.

Second, the findings also provide a challenge to the proposal that “embodied” processes play a causal role in visual perspective taking. Several recent studies have revealed that, to take another’s visual perspective, people have to mentally rotate their own body into the location and orientation of the other person, with time taken to judge another’s perspective increasing the more this person is rotated away from oneself (Kessler & Rutherford, 2010; Kessler & Thomson, 2010; Kozhevnikov et al., 2006; Surtees et al., 2013). Importantly, our data do not argue against such a mental rotation process *per se*. They do suggest, however, that this process is not *initiated* motorically, as this would have been affected by a person’s perceived ability to move, or their ability to make active body movements to trigger forward modelling of the anticipated sensory input if this mental rotation into the other’s body were completed (Decety, 1995; Moreau, 2013; Parsons, 1994; Wohlschläger & Wohlschläger, 1998). Instead, our findings are more consistent with the idea that shifts into the other’s perspective emerge from a purely visual transformation into the other’s space. The body movements of participants that we sometimes observed in our task are therefore more likely to reflect an epiphenomenal “leakage” of these simulated changes in viewpoint, as is typically observed for other forms of imagined action (e.g., Bach et al., 2010; Colton et al., 2018; Jacobson, 1930).

It is important to note that while we do anecdotally report that participants moved their bodies when they were free to do so, we did not measure the body movements they did make, nor did we include a condition in the present study, in which participants were actively instructed to move. We can therefore neither test in the current study to what extent such body movements are stronger in participants with a stronger tendency to perspective-take, nor whether actively inducing body movements that are congruent or incongruent with the other person’s location would help or hinder visual perspective taking (as seen, for example, in Kessler & Thomson, 2010). Instead, our data provide direct evidence that the (in-)ability to make these movements (in the no-movement condition) does not disrupt simulated changes in viewpoint. They therefore suggest that, while visual perspective taking may depend on the ability to *mentally* plan or imagine such bodily transformations through space, it does not depend on the ability to *physically make* these movements to trigger shifts into the others’ perspective (see Bach Allami, Tucker & Ellis, 2014, for a similar argument for motor imagery of manual actions). If this is correct, the inadvertent body movements we sometimes observe in our task are better explained through an inverse planning process that derives (and sometimes inadvertently elicits) body movements that realise the imagined spatial transformations, rather than forward modelling processes that predict the visuospatial transformations that happen as a consequence of the body movements one makes (see Bach, Fenton-Adams & Tipper, 2014; Colton et al., 2018; Wohlschläger, 2000 for a similar argument for motor imagery of manual actions).

A second, more exploratory, goal of the study was to determine whether individual differences are related to the stronger (or weaker) tendencies to take another’s visual perspective across participants, given that perspective-taking and mentalising more generally has been linked to an individuals’ social interaction abilities (e.g., Batson et al., 1997; Erle & Topolinski, 2015; Tomasello et al., 2005) and their breakdown in conditions such as schizophrenia (e.g., Langdon & Coltheart, 1999, 2001; Mohr et al., 2006) or autism spectrum conditions (Frith et al., 1991; Hamilton et al., 2009; for a review, see Hamilton, 2009). To this end, we correlated our individual measures of perspective taking with common individual difference measures that have been empirically or conceptually linked to perspective taking and Theory of Mind, such as schizotypal traits (STQ; Claridge & Broks, 1984), autistic traits (AQ; Baron-Cohen et al., 2001), or the ability to coordinate social interactions (IRI; Davis, 1983). Our data replicate the existing (but relatively weak) link between schizotypal symptoms and problems with taking others’ perspective (Langdon & Coltheart, 2001; Langdon et al., 2001). Measures of social ability and empathy (AQ and IRI), however, did not correlate with perspective taking, even when individual subscales were considered, and Bayesian analyses provided considerable evidence *against* such a link.

These findings may be surprising in light of the proposed link between visual perspective taking and mentalising and other coordination processes in social interactions (e.g., Batson et al., 1997; Bukowski & Samson, 2017; Erle & Topolinski, 2015; Freundlieb et al., 2016, 2017; Mattan et al., 2016; Tomasello et al., 2005) and that those with autism find it more difficult to make perceptual judgements from another’s visual perspective (Hamilton et al., 2009; for a review, see Pearson et al., 2013). Of course, our task involved people with autistic traits only, without actual diagnoses of autism spectrum disorder (ASD). Moreover, it did not involve actual social interactions, so that any fundamentally “social” mechanisms may not be engaged as effectively. Nevertheless, even in the prior literature, such relationships are inconsistent, and often found most robustly in children but not adults with ASD, implying that, while perspective taking might be delayed in ASD, it may have mostly caught up when participants reach adulthood, such as in the present sample. In addition, difficulties with representing another’s view in ASD are usually observed in tasks in which people must explicitly take others’ perspectives (Pearson et al., 2013), but less so in more implicit tasks such as ours (e.g., Zwickel et al., 2010). This is consistent with the proposal that those with ASD may have primarily problems with intentionally *selecting* one of several possible perspectives (Qureshi et al., 2010; Ramsey et al., 2013; Schwarzkopf et al., 2014) instead of the spontaneous *computation* of such perspectives, which is measured by our task.

If these considerations are taken seriously, then our data are more consistent with the view that perspective taking may not have specifically developed—either in ontogeny or phylogeny—to support social interactions but may build upon a more fundamental process of navigation and action planning (e.g., Kozhevnikov et al., 2006; Quesque et al., 2020; Ward et al., 2020). To effectively act in the world, humans constantly need to be able to derive from which location they may be able to see an object clearly or operate on it effectively. Visual perspective taking may have developed from this basic skill to imagine the world from another location that one could occupy, with other people providing simple landmarks to drive these processes. Several findings seem to support such an account. For example, it has been known for a long while that people’s ability to take another’s perspective is correlated with navigation skills (Allen et al., 1996; Hegarty & Waller, 2004; Kozhevnikov et al., 2006) and it has been reported that people are as ready to view the world from another person’s perspective as from the perspective of a landmark that supports navigation towards it, such as an empty chair (Gunalp et al., 2019; Quesque et al., 2020). In neuroimaging studies of mentalising and perspective taking (see Bukowski, 2018 for a critical review; Schurz et al., 2013 for meta-analysis), key regions such as the temporoparietal junction (TPJ) and the precuneus are also implicated in (imagined egocentric) navigation (Boccia et al., 2014, 2017; Committeri et al., 2015), and (virtual) lesion of the TPJ in particular can induce out-of-body experiences (Blanke et al., 2005), effectively moving oneself mentally into other possible locations one could occupy. In our own work with the present task, we have found that perspective taking is not sensitive to “social” features of the other person, such as whether they are looking at the item to be judged or not (Ward et al., 2020), but specifically their location in space, also in line with a social process of perspective taking that builds upon more fundamental navigational abilities.

If these links were borne out by future research, it may suggest that at least the spontaneous shifts of perspective measured in our and the above tasks rely on fundamental spatial abilities, which put one into another’s shoes, but do not necessarily let one see through their eyes. Future studies should include measures of navigational skill in perspective taking tasks. When these abilities are properly accounted for, and parcelled out, it may be possible to uncover the more social components that drive perspective taking. It may then be possible to describe not only how visual perspective taking has developed out of basic skills for spatial navigation, but also how more sophisticated processes for mentalising and Theory of Mind build upon these processes, to help us understand other people better and interact with them more effectively.

## Conclusion

Our results confirm, first, that people represent others’ viewpoints in a quasi-perceptual manner, such that other’s perspectives can “stand in” for own input and drive subsequent item recognition and mental rotation processes. Second, they show that people can derive others’ visual perspectives irrespective of whether they could move their own bodies, ruling out that physical movement is necessary either to trigger “embodied” perspective taking processes or to physically align one’s own perspective with that of the other person. Third, people’s spontaneous tendency to perspective take is negatively linked to their schizotypal traits but increases with age. It is, however, relatively independent from their autistic traits and their competency in coordinating social interactions, pointing towards a reliance on more fundamental processes of mental travel and imagined navigation.

## Supplemental Material

sj-docx-1-qjp-10.1177_17470218221077102 – Supplemental material for Is implicit Level-2 visual perspective-taking embodied? Spontaneous perceptual simulation of others’ perspectives is not impaired by motor restriction Click here for additional data file. Supplemental material, sj-docx-1-qjp-10.1177_17470218221077102 for Is implicit Level-2 visual perspective-taking embodied? Spontaneous perceptual simulation of others’ perspectives is not impaired by motor restriction by Eleanor Ward, Giorgio Ganis, Katrina L McDonough and Patric Bach in Quarterly Journal of Experimental Psychology
