# Supplementary material for: Is implicit Level-2 visual perspective-taking embodied? Spontaneous perceptual simulation of others’ perspectives is not impaired by motor restriction
Source: Q J Exp Psychol (Hove). 2022 Feb 25;75(7):1244–58. doi: 10.1177/17470218221077102 (PMC9131407; doi:10.1177/17470218221077102)
Supplement: sj-docx-1-qjp-10.1177_17470218221077102 – Supplemental material for Is implicit Level-2 visual perspective-taking embodied? Spontaneous perceptual simulation of others’ perspectives is not impaired by motor restriction [file sj-docx-1-qjp-10.1177_17470218221077102.docx]

Supplementary Material for:

**Is Implicit Level-2 Visual perspective taking embodied? Spontaneous perceptual simulation of others’ perspectives is not impaired by motor restriction.**

Eleanor Ward, Giorgio Ganis, Katrina L. McDonough, Patric Bach

| **Supplementary Table.** IRI, AQ, and STQ scores, age, handedness (left, right), gender (male, female), dyslexia diagnosis (yes, no), ASD diagnosis (yes, no). Correlations (*r*) with perspective taking score **p*<.05. ***p*<.01, ****p*<.001 | | | | | | | | | |
| --- | --- | --- | --- | --- | --- | --- | --- | --- | --- |
| Variables | 1 | 2 | 3 | 4 | 5 | 6 | 7 | 8 | 9 |
| 1. Perspective taking score | - |  |  |  |  |  |  |  |  |
| 1. AQ score | .08 | - |  |  |  |  |  |  |  |
| 1. IRI score | .03 | -.36** | - |  |  |  |  |  |  |
| 1. STQ score | -.26* | .19 | .05 | - |  |  |  |  |  |
| 1. Age | .38** | .24 | -.08 | -.06 | - |  |  |  |  |
| 1. Handedness | .07 | .18 | -.01 | .09 | .04 | - |  |  |  |
| 1. Gender | .10 | -.05 | .22 | -.18 | -.18 | -.07 | - |  |  |
| 1. Dyslexia | -.17 | .02 | -.28* | -.1 | -.03 | .38** | -.4** | - |  |
| 1. ASD | .11 | .07 | .05 | -.09 | .12 | .57*** | .07 | -.02 | - |

^6.^ coded as 1=left and 0=right ^7.^coded as 1=female, 0=male ^8.^coded as 1=yes, 0=no, ^9.^coded as 1=yes, 0=no.
